# The Infinitely Small
*Researches on Micro-Organisms; including an account of Recent Experiments on the Destruction of Microbes in Certain Infectious Diseases; Phthisis, &c. By A. B. Griffiths, Ph.D., F.R.S.E., F.C.S., Author of "The Diseases of Crops," &c. London: Ballière, Tindal, and Cox, 20, King William Street, Strand.


**Published:** 1891-06-27

**Authors:** 


### The Infinitely Small.*

Dr. Griffiths' volume on micro-organisms is as complete and compact as could be desired. It gives an account of almost every microbe that has yet been discovered, with instructions how to cultivate, study, and destroy it. Admirable diagrams are given of the necessary furnishings of the bacteriologist's laboratory, and historically, biologically, and chemically, the microbe has everything done for him. Dr. Griffiths is in deed an enthusiast in the pursuit of the bacillus, and though, as he says, "neither a physician nor a surgeon," he has done work that will be profitable to both. Naturally, it is as a scientist, an investigator in microscopic biology, that his work is most convincing and satisfactory; and many of his readers will gladly accept his researches without adopting all the conclusions he comes to in those departments which he expressly avows he does not profess. The medical profession owes a deep debt to bacteriology, and will gladly admit it, though perhaps it will not wholly endorse Dr. Griffiths' boast that the new study is destined to "raise medicine from empiricism to an exact science." Dr. Griffiths has the single-mindedness of the enthusiast; to him the microbe is all and in all; and though he quotes with approval, we doubt if he altogether realises the importance of Koch's warning as to the circumstances under which alone a microbe may be regarded as the exciting cause of disease. To make sure of this, the microbes taken from the body of any animal which has died of the disease under investigation must be cultivated with all possible precautions as to purity, then injected into the body of some other animal, where it must produce the characteristic symptoms and lesions of the disease, and multiply itself in so doing. The mere presence of microbes of similar character in different cases of the same disease proves nothing, for they may be of the most harmless character—innocuous microbes being, after all, far more numerous than dangerous ones.

This is not generally understood. The mystic word "bacteria" has been hurled at the popular head with so much emphasis and so little explanation that many people live under the apprehension of minute "fiery serpents," who are always on the look-out for victims, and crawl about us day and night. We have known people feel comforted by the knowledge that microbes, harmless or the reverse, are vegetal rather than animal in composition. It took away the element of repulsiveness that arose from the notion of the bacillus as being either parasite or entozoon. How small a thing a microbe is it is not easy for the average mind to realise. Dr. Griffiths attempts an estimate by a bold figure when speaking of the magnifying power (3,000 to 4,000 diameters) of the Geiss microscope: "Could we view a man under such a lens he would appear from three to four miles in height, or as high as Mount Blanc, Mount Ararat, or even Chimborazo. But even under this colossal magnification the smallest microbes do not appear larger than the points and commas of ordinary print." To estimate the number of creatures so infinitely small that may be found in a cubic inch of air or an ounce of water would tax one's arithmetic to the utmost, and the knowledge that even a small proportion of the millions of active bodies that surround us are pathogenic, or disease-producing, is sufficiently alarming. They are increased, if not generated, by the presence of man; most numerous in crowded houses and thoroughfares, they are rare on mountain-tops, and are not to be found at sea at a distance of 120 miles from land. It is curious to learn that microbes are absent from the air of sewers, being there absorbed by the sewage waters. Dr. Griffiths warns us, however, that as a very slight disturbance of this water may convey small liquid particles charged with micro-organic life to a considerable distance, "it must not be too hastily concluded, because sewer air is generally remarkably free from microbes that, therefore, a visit to the sewers should be attended with such beneficial results as a trip to the sea or the ascent of a mountain summit."

One of the most interesting chapters in Dr. Griffiths' book, certainly the one that is most likely to lead to controversy, is that in which he deals with the immunity from disease to be obtained by inoculation. He is a Pasteurian of the most thorough-going school, and puts aside with scorn the suggestion that Pasteur has "cured" people of hydrophobia who never suffered from it. "The same objection," he says, "was urged against Sir Spencer Wells, Mr. Lawson Tait, Dr. Keith, and others; and yet it must have been very difficult for them to invent ovarian tumours." Very difficult indeed; but the cases are not parallel. The first ovariotomy was performed on a woman who was obviously suffering from a tumour of some kind; the earliest operator knew this, even if he did not know that it was the ovary that was affected. He dealt with the ascertained fact of tumour; M. Pasteur's misfortune is that he has to deal, before the event, with a possible case of hydrophobia. This must always discount his results, until indeed he saves the life of a patient in whom the symptoms of hydrophobia have already appeared. Pasteur's method cannot be explained, like Jenner's, on the simple theory of exhaustion—that the introduction of the artificially cultivated and attenuated virus exhausted the soil on which the stronger poison might have seized. The notion that the weak virus produces by its action an antidote to its own stronger form is exceedingly difficult of acceptance, and Dr. Griffiths brings forward no satisfying proof. He quotes, with apparent approval, the theory of Dr. Gamaleia, of Odessa, as to the action of Pasteur's inoculations, that "the amœboid white blood corpuscles absorb and digest these live germs, and their power of absorption for germs is trained and increased by the progressively stronger inoculations, so that finally the virus deposited by the rabid animal can also be absorbed and destroyed." In short, "*l'appétit vient en mangeant.*" Very different from this is Koch's theory that the bacillus once introduced into the system devours all the tissue suited to its constitution, and then dies of starvation. His idea of protective inoculation is the introduction of a virus that will absorb tissues already decayed, without having strength to attack that which is still healthy, and will then die off. Where bacteriologists differ so widely it is rash to suggest any compromise. No satisfactory theory of the process of inoculation with the virus of a disease from which the patient is already suffering has yet been formulated, but if the practical results were what they could desire plain people would be content to let theories alone. At the same time, we must say that Dr. Griffiths does not argue, but simply begs, the question when he says that "perhaps the most important fact which proves the value of the Pasteurian method of inoculation is that 'Pasteur Institutes' are now established in Odessa, St. Petersburg, Rio Janeiro, Moscow, Buenos Ayres, Constantinople, Palermo, Naples, Havannah, and other places." Let us have clear statistics as to the results obtained in these institutes before we are asked to accept them as proving anything. Dr. Griffiths is a careful observer, a conscientious experimenter, but in his arguments in favour of inoculation he does not impress us as being a logical thinker.

We must acknowledge, however, the great value of the life-histories he brings together with a view to showing the infectious nature of consumption. Most people admit that phthisis is hereditary, but it cannot be too generally impressed on the public that it is also highly contagious, very often passing from husband to wife, and *vice versa*, and often affecting members of the same family, not because of any hereditary taint, but through the simple fact of close companionship. On this point Dr. Griffiths says nothing original, but he quotes a number of cases which are calculated to make his readers think. Indeed, throughout his whole volume, there is little claim to originality save in the discovery of microbes and their habits; but as a collection of the ascertained facts on the subject it forms a useful contribution to bacteriology.

* Researches on Micro-Organisms; including an account of Recent Experiments on the Destruction of Microbes in Certain Infectious Diseases; Phthisis, &c. By A. B. Griffiths, Ph.D., F.R.S.E., F.C.S., Author of "The Diseases of Crops," &c. London: Ballière, Tindal, and Cox, 20, King William Street, Strand.

